# Cyclooxygenase-2 Upregulated by Temozolomide in Glioblastoma Cells Is Shuttled In Extracellular Vesicles Modifying Recipient Cell Phenotype

**DOI:** 10.3389/fonc.2022.933746

**Published:** 2022-07-22

**Authors:** Francesca Lombardi, Francesca Rosaria Augello, Serena Artone, Emira Ayroldi, Ilaria Giusti, Vincenza Dolo, Maria Grazia Cifone, Benedetta Cinque, Paola Palumbo

**Affiliations:** ^1^ Department of Life, Health and Environmental Sciences, University of L’Aquila, L’Aquila, Italy; ^2^ Department of Medicine and Surgery, Section of Pharmacology, University of Perugia, Perugia, Italy

**Keywords:** extracellular vesicles, glioblastoma, temozolomide, COX-2, COXIB, celecoxib, NS398

## Abstract

Temozolomide (TMZ) resistance is frequent in patients with glioblastoma (GBM), a tumor characterized by a marked inflammatory microenvironment. Recently, we reported that cyclooxygenase-2 (COX-2) is upregulated in TMZ-resistant GBM cells treated with high TMZ concentrations. Moreover, COX-2 activity inhibition significantly counteracted TMZ-resistance of GBM cells. Extracellular vesicles (EV) are considered crucial mediators in orchestrating GBM drug resistance by modulating the tumor microenvironment (TME) and affecting the surrounding recipient cell phenotype and behavior. This work aimed to verify whether TMZ, at low and clinically relevant doses (5-20 µM), could induce COX-2 overexpression in GBM cells (T98G and U87MG) and explore if secreted EV shuttled COX-2 to recipient cells. The effect of COX-2 inhibitors (COXIB), Celecoxib (CXB), or NS398, alone or TMZ-combined, was also investigated. Our results indicated that TMZ at clinically relevant doses upregulated COX-2 in GBM cells. COXIB treatment significantly counteracted TMZ-induced COX-2 expression, confirming the crucial role of the COX-2/PGE2 system in TMZ-resistance. The COXIB specificity was verified on U251MG, COX-2 null GBM cells. Western blotting of GBM-EV cells showed the COX-2 presence, with the same intracellular trend, increasing in EV derived from TMZ-treated cells and decreasing in those derived from COXIB+TMZ-treated cells. We then evaluated the effect of EV secreted by TMZ-treated cells on U937 and U251MG, used as recipient cells. In human macrophage cell line U937, the internalization of EV derived by TMZ-T98G cells led to a shift versus a pro-tumor M2-like phenotype. On the other hand, EV from TMZ-T98G induced a significant decrease in TMZ sensitivity in U251MG cells. Overall, our results, in confirming the crucial role played by COX-2 in TMZ-resistance, provide the first evidence of the presence and effective functional transfer of this enzyme through EV derived from GBM cells, with multiple potential consequences at the level of TME.

## Introduction

Extracellular vesicles (EV), a heterogeneous population of lipid bilayer-enclosed structures released from all cell types, have been defined as critical mediators of intercellular communication by transferring functional genomic and proteomic cargo. The molecular content of EV, namely proteins, nucleic acids, and lipids, reflects the status and phenotype of the releasing cells. The EV are essential actors in the tumor microenvironment (TME), including glioblastoma (GBM), the most common and aggressive type of primary intracranial tumor in humans, representing about 81% of the malignant oncological lesions of the brain (1). Nowadays, although remarkable advances in GBM therapy have driven significant progress, chemoresistance remains the main hurdle in patient survival. Temozolomide (TMZ) is an oral DNA alkylating agent currently used as a standard first-line treatment for adult patients affected by newly diagnosed GBM. This drug exerts its antitumor activity by interfering with DNA replication. TMZ methylates DNA leading to the formation of O6-methylguanine, the most potent cell-killing lesion, which mispairs with thymine during the next cycle of DNA replication. Although TMZ can improve the overall survival of patients, the therapeutic outcomes remain unsatisfactory (2).

The ability of GBM cells to dynamically modulate the EV cargo composition in response to chemotherapy and hypoxia is becoming increasingly evident; on the other hand, how the EV cargo can affect the target cell phenotype, modifying sensitivity to chemotherapy drugs, thus promoting chemoresistance (3–5), is currently being studied.

The combined use of the chemotherapeutic agent TMZ with COX-2 inhibitors (COXIB) has been investigated as an alternative strategy to fight GBM progression by counteracting chemoresistance (6). As the proinflammatory enzyme COX-2 is recognized as a crucial mediator in GBM biology, selective COXIB were defined as an extremely promising GBM therapy increasing sensitivity to chemotherapy without other side effects (7). In this regard, recently, our group reported that the selective COX-2 inhibitor, NS398, counteracted chemoresistance to TMZ, used at high concentrations (200 µM) for 72 h, in resistant GBM cell line (T98G) abrogating TMZ-induced COX-2 upregulation and COX-2-dependent pathways involved in TMZ-resistance (8). TMZ exposure of resistant T98G, but not sensitive and COX-2 null U251MG cells, led to a significant and dose-dependent upregulation of COX-2. Moreover, NS398 enhanced the chemosensitivity to TMZ in GBM cells downregulating TMZ-induced COX-2 expression. The ability of Celecoxib (CXB), a selective COXIB approved by the Food and Drug Administration (FDA) and widely used for its anti-inflammatory, analgesic, and antipyretic actions, to suppress the growth of GBM cell lines, U373 and T98G, partly by inhibiting the NF-kB signaling pathway, has also been reported (9). A recent *in vitro* study highlighted the CXB ability combined with TMZ at a high concentration (250 µM) to reverse chemoresistance of TMZ-resistant GBM cell lines, LN229 and LN18, affecting cell proliferation and inducing apoptosis and autophagy by the inhibition of the mitochondrial metabolism and respiratory chain (10). Also, CXB has been studied in several clinical trials in combination with other drugs such as TMZ (11, 12). In general, the results from these *in vivo* studies support the potentially effective use of low-dose metronomic CXB combined with TMZ in treating GBM patients not eligible for standard treatment (13–15).

In this study, the first aim was to analyze the effect of TMZ, at clinically relevant concentrations (5-20 μM), on T98G ability to release EV when daily exposed to long-term treatment (5 days), a condition conceived to mimic the clinical and therapeutical setting, during which intratumoral TMZ concentrations between 1 and 35 µM are achieved (16). Thus, to improve the transferability of *in vitro* results to *in vivo* studies, we initially evaluated whether TMZ at clinically relevant concentrations could induce a cytotoxic effect and a significant increase of COX-2 level similar to what we previously registered with TMZ at higher concentrations in T98G cells, chosen as TMZ-resistant/COX-2 positive cell line (8). The U251MG cells were used as a negative control, being TMZ-sensitive andCOX-2 null. Also, we preliminarily verified whether COX-2 inhibition could counteract the resistance of T98G cells exposed to TMZ. COX-2 levels were evaluated in EV secreted by T98G and U87MG cells after the scheduled treatment program with drugs alone or in combination. COX-2 over-expression or exogenous PGE2 had been reported to promote the macrophage polarization to M2 phenotype in breast cancer (17). Also, the COX-2 inhibition caused the loss of M2 features and suppressed the tumor metastasis (18). Based on these findings, we have investigated the effects of EV released by T98G cells after treatments on the phenotype of macrophages used as recipient cells. Moreover, the ability of the EV released by TMZ-treated T98G cells to affect TMZ-sensitivity was evaluated in U251MG recipient cells.

## Materials and methods

### Cell Culture and Treatments

Human GBM cell lines T98G and U87MG were acquired from the European Collection of Authenticated Cell Cultures (ECACC) and U251MG cell line was acquired from Cell Lines Service (CLS). Cells were cultured using manufacturer recommendations in Dulbecco’s Modified Eagle’s Medium (DMEM) supplemented with 10% (v/v) of fetal calf serum (FCS), 2 mM L-glutamine, 100 U/ml penicillin, and 100 mg/ml streptomycin (complete medium) (EuroClone, West York, UK) at 37°C in 5% CO_2_ and 95% humidity and media was totally replaced every 3 days. Human monocyte cell line, U937, widely used in *in vitro* experiments as a human macrophage model (19, 20), was acquired from Cell Lines Service (CLS) and cultured in RPMI-1640 medium (EuroClone, West York, UK) supplemented with 10% (v/v) of FCS, 2 mM L-glutamine, 100 U/ml penicillin, and 100 mg/ml streptomycin (complete medium) in a 5% CO_2_ humidified atmosphere at 37°C.

T98G was chosen as chemoresistant cell line for TMZ, displaying a LC50 ranging from >250 mM to 1585 mM, U87MG as chemosensitive cell line for TMZ, showing a LC50 ranged into 7 μM to 172 μM and U251MG cells as chemosensitive cell line, showing a LC50 around 50 µM (21). From the temozolomide (TMZ, Sigma-Aldrich, Saint Louis, MO, USA) stock solution (51.5 mM in dimethyl sulfoxide, DMSO, which had no significant effect on treated cells), the concentrations of 5, 10, and 20 µM were daily added to cells, initially plated at 3.5 × 10^3^ cells/cm^2^, for 5 days. Based on previous reports (22), the Celecoxib (CXB) concentration of 8 μM was used (Sigma-Aldrich, Saint Louis, MO, USA). NS398 (N-[2-(Cyclohexyloxy)-4-nitrophenyl] methanesulfonamide) (23) (Sigma-Aldrich, Saint Louis, MO, USA), was stored as stock solutions in DMSO at −20°C according to the manufacturer instruction and diluted in cell culture medium just before use at the low concentration of 20 μM for the indicated times. In order to assess the effect of TMZ alone or in combination with CXB or NS398 on GBM cells and on the GBM-EV content, the cells were plated for all experiments in 25 cm^2^ plates at 3.5 × 10^3^ cells/cm^2^, left to adhere and then daily treated or not (CNTR) with TMZ, CXB or NS398, at selected concentrations alone or in combination. The treatment with the DMSO alone (vehicle) was referred throughout the manuscript as “control” (CNTR).

To mimic the clinical condition as much as possible, the exposure to TMZ and the combined treatment schedule with CXB or NS398, were repeated daily for 5 days (referred as a “long-term exposure”) (24). To analyze cell viability following treatments, the 0.04% Trypan blue (EuroClone, West York, UK) solution was used. The cells were transferred to a Bürker counting chamber and then counted by microscopy (Eclipse 50i, Nikon Corporation, Tokyo, Japan). The GBM cell lines morphology was visualized and imaged by Nikon Eclipse TS100. Where not otherwise specified, the reagents and consumables were purchased from EuroClone (EuroClone, West York, UK). All cell lines were routinely tested for mycoplasma and were negative prior to use.

### Extracellular Vesicle Isolation

To isolate EV, the cells were cultured in complete medium, replacing the FCS with Hyclone 40 nm filtered serum (Thermo Scientific, Rockford, IL, USA) and supernatants collected after treatments were centrifuged at 600×g for 15 min and then at 1500×g for 30 min at 4°C to remove cells and large debris, respectively. The resulting supernatants were then centrifuged at 100,000×g for 90 min at 4°C in an Optima XPN-110 Ultracentrifuge Rotor 70Ti, Quick-Seal Ultra-Clear tubes, k_adj_ 197, brake 9 (Beckman Coulter, CA, USA). Isolated EV were resuspended in Dulbecco’s phosphate-buffered saline (PBS) (EuroClone, West York, UK) according to proper dilutions, and the determination of their quantification was carried out by measuring the vesicle-associated protein levels using DC Protein Assay (Bio-Rad, Hercules, CA, USA) using BSA as standard.

### Transmission Electron Microscopy

Transmission electron microscopy (TEM) was performed on EV isolated as described above. To this aim, after collection, EV, resuspended and diluted in PBS according to proper dilutions, were adsorbed onto 300-mesh carbon-coated copper grids (Electron Microscopy Sciences, Hatfield, PA, USA), fixed in 2% glutaraldehyde (Electron Microscopy Sciences) in PBS for 10 min, rinsed in Milli-Q water, and negative stained with 2% phosphotungstic acid. Grids were examined with a Philips CM 100 transmission electron microscope TEM (Philips, Eindhoven, Netherlands).

### NanoSight

EV number and size were assessed by the nanoparticle tracking analysis (NTA). Using a NanoSight NS300 (NanoSight Ltd., Amesbury, UK), EV were visualized by laser light scattering. Briefly, EV-enriched pellets were resuspended in sterile PBS to generate a proper dilution and five recordings of 60 sec were performed for each sample; 1498 frames in total were examined, captured, and analyzed by applying optimized settings. Data were analyzed with the NTA software, which provided the concentration measurements (particles/ml) and size distribution profiles for the EV in solution.

### Protein Extraction and Western Blotting Assay

GBM cell pellets were homogenized and lysed in ice-cold RIPA buffer (phosphate buffer saline pH 7.4) (Merck KGaA, Darmstadt, Germany) supplemented with 100 mM protease inhibitor cocktail (Sigma-Aldrich, Saint Louis, MO, USA). Protein lysates (25 μg/lane) were separated on 10% SDS–polyacrylamide gel under reducing conditions with β-mercaptoethanol 5% and electroblotted onto 0.45 µm nitrocellulose membrane sheets (Whatman-GE Healthcare Life Sciences, UK). To remove non-specific binding sites, membranes were incubated with 5% non-fat dry milk in Tris buffered saline for 1 h at room temperature and then incubated overnight at 4°C with primary antibodies: rabbit monoclonal anti-COX-2 (Cell Signaling Technology, Danvers, MA, USA; dilution 1:1000), mouse monoclonal antibody anti-MGMT (BD Biosciences, San José, CA, USA; dilution 1:500), rabbit polyclonal anti-β-catenin antibody (Cell Signaling Technology, Danvers, MA, USA; dilution 1:1000), and mouse monoclonal antibody for anti-β-actin (Bio-Rad, Hercules, CA, USA; dilution 1:1000). As secondary antibodies, peroxidase conjugated anti-rabbit and anti-mouse IgG antibodies (dilution 1:2000) were acquired from Sigma-Aldrich (Saint Louis, MO, USA).

For Western blotting on EV lysate proteins (10 μg/lane) were resolved on 10% SDS–polyacrylamide gel electrophoresis under non-reducing conditions with heating (CD63, TSG101) or without heating (CANX) and blotted to nitrocellulose membranes (Whatman-GE Healthcare Life Sciences, UK); blocking was performed for 90 min in 10% non-fat dry milk in TBS containing 0.5% Tween-20 (TBS-T) at room temperature. The blots were then incubated at 4°C overnight with primary antibodies diluted in TBS-T containing 1% non-fat dry milk: rabbit polyclonal anti-CANX antibody (Immunological Sciences, Italy; dilution 1:1000), mouse monoclonal anti-CD63 (Santa Cruz Biotechnology Inc, Dallas, TX, USA; dilution 1:400), rabbit polyclonal anti-TSG101 (Immunological Sciences, Italy; dilution 1:2000). The membranes were washed in TBS-T and incubated for 1 h at room temperature in a peroxidase-conjugated secondary antibody diluted in TBS-T containing 1% non-fat dry milk (goat anti-mouse IgG-HRP, 1:10,000 dilution; goat anti-rabbit IgG-HRP, 1:7500 dilution; Santa Cruz Biotechnology, Inc.).

Chemiluminescent detection was performed using the ECL (Amersham Pharmacia Biotech) according to the manufacturer’s instructions. Emission was captured using the chemiluminescence documentation system ALLIANCE (UVITEC, Cambridge UK). For EV proteins, the relative protein levels were calculated based on GAPDH (OriGene, Rockville, MA, USA; dilution 1:1000) as the loading control.

### Prostaglandin E_2_ (PGE_2_) Level Assay

The levels of secreted PGE_2_ were measured in supernatants of GBM cells daily exposed or not (CNTR) for 5 days to TMZ, CXB, or NS398, as described above. The supernatants were then assayed for prostaglandin E_2_ (PGE_2_) levels by an enzyme-linked immunosorbent assay (ELISA) kit (Cayman Chemical Company, Ann Arbor, MI, USA). Results are presented as fold increase of released PGE_2_ vs. CNTR.

### Evaluation of Growth of U251MG Recipient Cells

The U251MG cells were seeded at 4x10^3^/cm^2^ and, once attached, were exposed to EV (30 µg/ml) derived from TMZ-treated T98G cells or untreated for 18 h to allow the internalization. Then, TMZ (10 µM) was added or not at culture media for 5 days, and cell growth was measured by Trypan blue staining as above described.

### Macrophage Polarization

The differentiation of U937, an oncogenic human monocyte cell line, into cells possessing a macrophage-like phenotype (M0) was achieved by exposure to 100 ng/ml of Phorbol-12-Myristate-13-Acetate (PMA; Sigma-Aldrich, Saint Louis, MO, USA) as previously reported (20, 25). After 48 h, the PMA-treated monocytes, referred to as “macrophage-like”, undergo a series of morphological and functional changes becoming adherent. The U937 were cultured in RPMI 1640 medium supplemented with 10% FBS, at a density of 1×10^4^ cells/ml in 6-well culture plates. Macrophage M2 polarization was obtained by incubation with 20 ng/ml of interleukin 4 (IL-4) (Peprotech, Rocky Hill, NJ, USA) and 20 ng/ml of interleukin 13 (IL-13) (Peprotech, Rocky Hill, NJ, USA) for additional 72 h.

### Extracellular Vesicles Labeling and Uptake of PKH26-Labeled T98G-EV by U937 Cells

To verify the uptake and target cell interaction of EV derived from T98G previously treated with CXB (8 μM), NS398 (20 μM), TMZ (10 μM) alone and in combination by U937 macrophagic cells, the fluorescent lipid membrane dye molecule PKH26 (PKH26 Red Fluorescent Cell Linker kit - Sigma-Aldrich, Saint Louis, MO, USA) staining was assessed according to manufacturer’s instructions. Briefly, the U937 cell line was grown on coverslips in a 12-well plate (seeded at 5×10^4^ cells/coverslips) and, once attached, was incubated in the presence or absence (CNTR) with EV derived from T98G treated as previously reported. For the PKH26 staining, the obtained EV were resuspended in 1 ml Diluent C. Then, 6 μL of PKH26 were added to each sample in sterile conditions. The EV suspension was mixed for 30 s with the stain solution and incubated for 5 min at room temperature. The labelling reaction was stopped by adding 2 ml of 10% BSA in sterile PBS. Labeled EV were ultracentrifuged as previously described. Negative technical control was made by adding the same volume of diluent C and PKH2 as samples. Afterward, U937 cells were incubated for 18 h at 37°C in a 95% air 5% CO_2_ atmosphere, with 30 µg PKH26-labeled EV derived from T98G previously treated with COX-2 inhibitors and TMZ alone and in their combinations. The coverslips were mounted with Vectashield^®^ Antifade Mounting Medium with DAPI (Vector Laboratories, Inc., Burlingame, CA, USA), and the effective EV internalization was observed by fluorescent microscopy (Nikon, Eclipse 50i, Tokyo, Japan). All images were acquired at 100×magnification.

### EV-COX-2 Immunofluorescence Staining

The U937 and the U251MG cells, both plated at 5×10^4^ cells/coverslips, were differently treated as above reported. For labeling, the coverslips were washed, fixed with 4% formaldehyde for 20 min, permeabilized with 0.1% Triton X-100 (Sigma-Aldrich, Saint Louis, MO, USA) for 5 min, and blocked with 3% BSA (Sigma-Aldrich, Saint Louis, MO, USA) for 20 min at room temperature. Cells were incubated overnight at 4°C with rabbit monoclonal anti-human COX-2 (Cell Signaling Technology, Danvers, MA, USA; dilution 1:400), and afterward with a FITC conjugated goat anti-rabbit polyclonal IgG secondary antibody (Millipore EMD, Darmstadt, Germany; dilution 1:1000) for 1 h at room temperature and washed. Coverslips were mounted with VECTASHIELD^®^ Antifade Mounting mounted Medium with DAPI (Vector Laboratories, Inc., Burlingame, CA, USA) and visualized as previously described.

### TGF-β1 ELISA

The levels of released TGF-β1 were quantified in the U937 cell supernatants using a human TGF-β1 enzyme-linked immunosorbent assay (ELISA) kit (Sigma Aldrich, Saint Louis, MO, USA), as described in the manufacturer’s instructions. Briefly, the U937 cells were plated at 1×10^5^ cells/ml and treated with EV derived from T98G previously daily exposed for 5 days with CXB, NS398, TMZ as single agent or in combination. The EV treatment lasted 72 h, then the media were collected, cleared of cellular debris/dead cells by centrifugation at 1000× g for 15 min, and the TGF-β1 concentration was then determined in the medium using the ELISA kit. Results are expressed as pg/ml.

### Statistical Analysis

Statistical analyses were performed using GraphPad Prism version 6.01, (GraphPad Software, San Diego, CA, USA). The data were evaluated using the one-way ANOVA test followed by Tukey or Dunnett’s *post hoc* test, where specified. Testing for synergistic or additive effects of combination therapy was performed according to Bliss independence analysis (26). Experiments were independently repeated three times at least and performed in duplicate or triplicate, and the results were shown as the means ± SEM (standard error mean). P-value <0.05 was considered to indicate a statistically significant difference.

## Results

### Effect of TMZ Alone or Combined With COXIB on GBM Cell Lines

**Figure 1:**
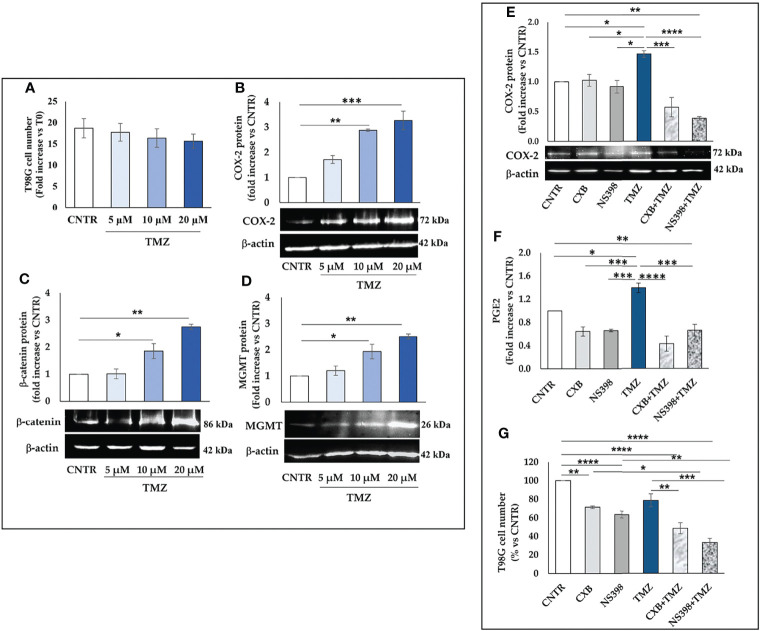
Influence of TMZ on T98G TMZ-resistant cells. Cells were daily incubated with increasing doses of TMZ (5-20 μM) for 5 days and **(A)** cell number was detected by Trypan blue staining. All values are given as fold increase vs. initial time (T0). The results, derived from three experiments performed in duplicate, are expressed as mean ± SEM. The one-way analysis of variance (ANOVA) followed by Dunnet *post hoc* test show not significant differences. Influence of TMZ on **(B)** COX-2, **(C)** β-catenin and **(D)** MGMT levels was assessed by Western blotting in the presence of vehicle (CNTR), or TMZ, as previously described. The obtained values were normalized vs. β-actin and presented as fold increase vs. CNTR. Data are from three independent experiments and values are expressed as mean ± SEM. For comparative analysis of data, a one-way ANOVA with Dunnet *post hoc* test was used (*P<0.05; **P<0.01; *** P<0.001 vs. CNTR). Representative images of each immunoblotting are shown. **(E)** Influence of the COXIB combined with TMZ on COX-2 levels was verified by Western blotting assay in T98G cells daily incubated or not (CNTR) with Celecoxib (CXB) (8 μM), NS398 (20 μM), TMZ (10 μM) or with the co-treatments (CXB+TMZ and NS398+TMZ) for 5 days. Densitometric analysis was performed by normalizing vs. β-actin and presented as fold increase vs. CNTR. Data from three independent experiments are expressed as mean ± SEM. Representative images of each immunoblotting are shown. C+ = positive control (not treated T98G cells). **(F)** PGE2 levels (fold increase vs. CNTR) released by T98G treated as above described, were assayed by ELISA kit. Results are expressed as mean ± SEM of three experiments in duplicates. **(G)** Effect of COXIB, CXB and NS398, TMZ and drug combinations on T98G cell number was evaluated by Trypan blue staining. All values are given as fold increase vs. CNTR of two independent experiments performed in triplicate (mean ± SEM). For comparative analysis of data groups, a one-way ANOVA with Tukey *post hoc* test was used (*P<0.05; **P<0.01; ***P<0.001; ****P<0.0001).

**Figure 2:**
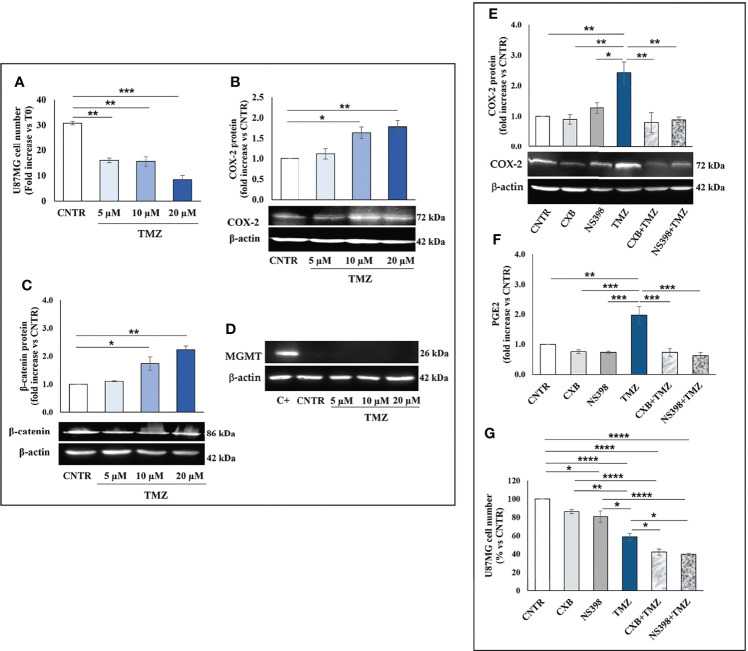
Influence of TMZ on U87MG TMZ-sensitive cells. Cells were daily incubated with increasing doses of TMZ (5-20 μM) for 5 days and **(A)** cell number was detected by Trypan blue staining. All values are given as fold increase vs. initial time (T0). The results, derived from three experiments performed in duplicate, are expressed as mean ± SEM. The one-way analysis of variance (ANOVA) followed by Dunnet *post hoc* test show not significant differences. Effect of TMZ on **(B)** COX-2, **(C)** β-catenin and **(D)** MGMT levels was assessed by Western blotting in the presence of vehicle (CNTR), or TMZ, as previously described. The obtained values were normalized vs. β-actin and presented as fold increase vs. CNTR. Data are from three independent experiments and values are expressed as mean ± SEM. For comparative analysis of data, a one-way ANOVA with Dunnet *post hoc* test was used (*P<0.05; **P<0.01; *** P<0.001 vs. CNTR). Representative images of each immunoblotting are shown. **(E)** Influence of the COXIB combined with TMZ on COX-2 levels was verified by Western blotting assay in U87MG cells daily incubated or not (CNTR) with Celecoxib (CXB) (8 μM), NS398 (20 μM), TMZ (10 μM) or with the co-treatments (CXB+TMZ and NS398+TMZ) for 5 days. Densitometric analysis was performed by normalizing vs. β-actin and presented as fold increase vs. CNTR. Data from three independent experiments are expressed as mean ± SEM. Representative images of each immunoblotting are shown. C+ = positive control (not treated T98G). **(F)** PGE2 levels (fold increase vs. CNTR) released by U87MG treated as above described, were assayed by ELISA kit. Results are expressed as mean ± SEM of three experiments in duplicates. **(G)** Effect of COXIB, CXB and NS398, TMZ and drug combinations on U87MG cell number was evaluated by Trypan blue staining. All values are given as fold increase vs. CNTR of two independent experiments performed in triplicate (mean ± SEM). For comparative analysis of data groups, a one-way ANOVA with Tukey *post hoc* test was used (*P<0.05; **P<0.01; ***P<0.001; ****P<0.0001).

TMZ affects the viability and proliferation of GBM cell lines when used at high concentrations (27, 28) while away from clinical practice. Thus, we firstly investigated the effect of a 5-day treatment with low clinically relevant concentrations of TMZ (5–20 μM) on the cell growth rate of T98G and U87MG. As expected, none of the TMZ tested concentrations significantly influenced the T98G cell number and no dead cell was detected at 5 days, confirming their high resistance (**Figure 1A**). The microscopy observation also supported these data. T98G cells exposed to low TMZ concentrations showed a cell density similar to control (CNTR) (**Supplementary Figure 1A**). We then evaluated the ability of TMZ to upregulate COX-2 in T98G cells treated as above described, through Western blotting (**Figure 1B**). TMZ incubation at 5 µM did not significantly affect the COX-2 expression compared to the CNTR sample, while the highest TMZ concentrations (10 and 20 µM) markedly influenced it. The levels of β-catenin and MGMT, two proteins strictly associated with COX-2 activity and strongly implicated in the GBM chemoresistance (29, 30), were also evaluated. The results showed that the expression of the β-catenin proportionally increased to TMZ concentrations being significant at 10 µM and 20 µM **(** **Figure 1C**). MGMT expression showed a similar trend (**Figure 1D**). Conversely, in the U87MG cell line, the daily treatment with TMZ at all concentrations reduced the cell proliferation rate evaluated at 5 days (**Figure 2A**), as confirmed by images taken by contrast-phase microscope (**Supplementary Figure 1B**). The levels of COX-2 and β-catenin were dose-dependently upregulated by TMZ exposure (**Figures 2B, C**). TMZ treatment did not induce MGMT expression in the U87MG cell line (MGMT negative) (**Figure 2D**). In line with our previous results (8), TMZ did not induce COX-2 expression, at all tested concentrations, in the TMZ-sensitive/COX-2 negative U251MG cells (31) (**Supplementary Figure 2A**), nor modulate β-catenin or induce the expression of MGMT (**Supplementary Figures 2B, C**, respectively).

To further elucidate the role of COX-2 in TMZ resistance, GBM cell lines were exposed to COXIB, alone or in combination with TMZ, after which the COX-2 expression and activity, as well as cell number, were evaluated. GBM cell lines were treated daily with CXB (8 μM), NS398 (20 μM), or TMZ (10 μM) alone or with their combinations (CXB+TMZ or NS398+TMZ) for 5 days. Representative Western blot images and the results from densitometric analysis of COX-2 levels in T98G cells are shown in **Figure 1E**. COXIB, used alone, did not influence the levels of COX-2. On the other hand, a significant increase of COX-2 was detected after treatment with 10 μM TMZ compared to CNTR. Of note, CXB or NS398 combined with TMZ significantly downregulated the TMZ-induced COX-2 (**Figure 1E**). PGE_2_ levels were directly measured in supernatants of cell cultures to evaluate the COX-2 activity and verify the specificity of COXIB. TMZ-treated cells released high amounts of PGE_2_, in line with the COX-2 upregulation. Both the drug combinations significantly reduced the COX-2 activity compared to TMZ alone (**Figure 1F**). A similar COX-2 expression and activity trend was observed in U87MG cells in which the TMZ-induced COX-2 upregulation was counteracted by drug combination treatments (**Figures 2E, F**). The protein was not expressed in the COX-2 null U251MG (**Supplementary Figure 2D** **).** Accordingly, PGE2 was undetectable in the U251MG culture media (not shown).

Next, we evaluated the effect of CXB, NS398, TMZ, and their combination on GBM cell number. After 5 days of continuous, scheduled treatment with COXIB, the T98G and U87MG cell number was decreased compared to CNTR cells (**Figures 1G**, **2G**). As previously reported, COX-2 inhibitors reduced the growth rate of GBM cell lines (8, 32). The reduced cell growth rate following CXB or NS398 treatment could be due to the inhibition of basal COX-2 activity and COX-2-dependent signaling pathways. Of note, both drug combinations, CXB+TMZ and NS398+TMZ, caused a statistically significant decrease in T98G and U87MG viable cell numbers compared to CNTR, TMZ alone, and relative COXIB alone (**Figures 1G**, **2G**). A Bliss independence test was performed to assess the nature of TMZ and COXIB interaction (synergistic or additive), suggesting that drug combination treatment had a synergistic effect on GBM cells vs. single agents.

### Upregulation of COX-2 in EV Derived from GBM Cells Treated with TMZ is Counteracted by COXIB

**Figure 3:**
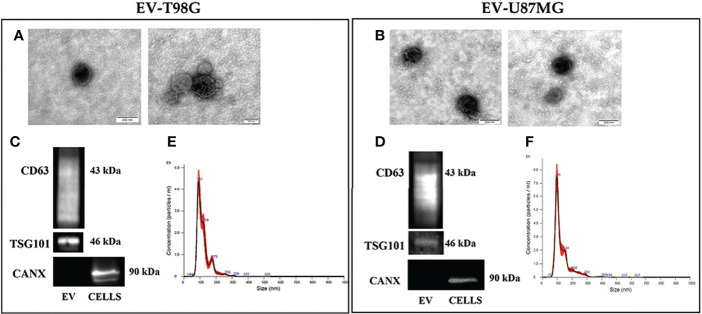
Characterization of EV population secreted from GBM cell lines, T98G and U87MG. **(A)** and **(B)** Representative electron microscopy images showing whole and round-shaped EV derived from T98G and U87MG, respectively. **(C)** and **(D)** Western Blot analysis of specific markers CD63, TSG101 and Calnexin on EV isolated from GBM cells. **(E)** and **(F)** Representative Nanoparticle Tracking Analysis (NTA) profiles of T98G-EV and U87MG-EV. Each curve was generated from five measurements.

**Figure 4:**
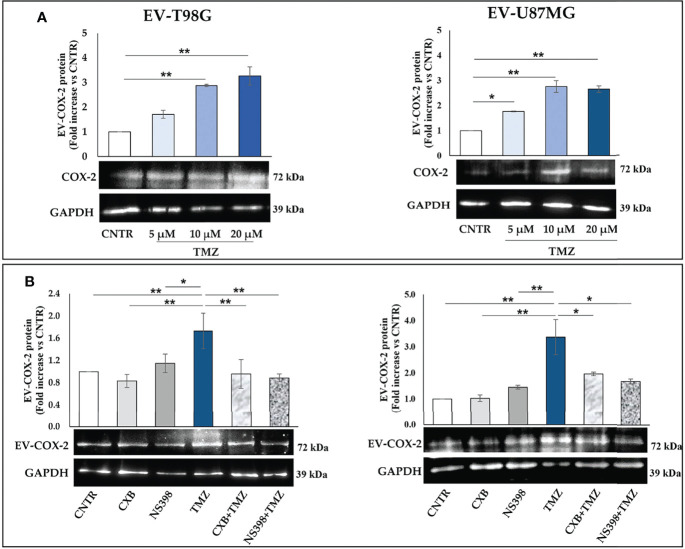
**(A)** COX-2 protein is transferred into EV derived by T98G and U87MG treated with TMZ in a dose-dependent way. GBM cells were daily exposed to increasing concentrations of TMZ (5–20 µM) for 5 days, EV were collected from cell supernatants and COX-2 levels were analysed by immunoblotting assay. Densitometric bands were normalized vs. GAPDH. Data from three independent experiments are shown as the mean ± SEM and expressed as fold increase vs. CNTR. Images from one representative out of three independent experiments are presented. For comparative analysis of groups of data, one-way ANOVA followed by Dunnett’s post hoc test was used (*P<0.05; **P<0.01 vs. CNTR). **(B)** COXIB, CXB or NS398, alone and in combination with TMZ, modulate the cargo of EV released from GBM cells. T98G and U87MG cell lines were daily exposed for 5 days to CXB (8 μM), NS398 (20 μM), TMZ (10 μM), alone or in combination and representative COX-2 and GAPDH immunoblots are shown. Following densitometric analysis, obtained values were normalized vs. GAPDH. Data are from three independent experiments, and values (mean ± SEM) are expressed as fold increase vs. CNTR. For comparative analysis of data, a one-way ANOVA with Tukey post hoc test was used (*P<0.05; **P<0.01).

We then investigated the effect of TMZ on the EV content of COX-2 in the T98G and U87MG cells. Firstly, the EV released from both GBM cell lines were collected and characterized according to MISEV (33) by TEM, Western blotting of specific markers, and NTA analyses. The TEM images showed T98G-EV and U87MG-EV with a round-shaped, membrane-enclosed structure (**Figures 3A, B**). Western blotting was performed to evaluate the expression of the specific EV markers, CD63 and TSG101, and to verify the absence of endoplasmic reticulum marker calnexin, indicating the EV purity without contamination of cell debris and organelles (**Figures 3C, D**). Isolated EV were then analyzed by NTA to determine the particle number and size distribution, displaying that most EV had a diameter less than 200 nm (*small vesicles*) (**Figures 3E, F**, respectively) (33). Next, T98G- and U87MG-derived EV were evaluated for the presence of COX-2 protein by Western blotting. The results evidenced that COX-2 protein was shuttled in EV of both cell lines, with levels dependent on TMZ concentrations, resulting significantly higher at 10 and 20 µM in T98G and at all tested concentrations in U87MG (**Figure 4A**). This trend was in line with COX-2 upregulation induced by TMZ in T98G (**Figure 1B**) and U87MG cells (**Figure 2B**).

Next, we evaluated whether COX-2 levels in EV released from T98G and U87MG exposed to low concentration of TMZ could be modulated by the concurrent treatment with CXB or NS398. The Western blot results of both cell lines showed a similar trend: the daily exposure to CXB and NS398 alone did not significantly modulate the content of COX-2 when compared to EV derived from CNTR (**Figure 4B**). On the other hand, an evident reduction of COX-2 in EV derived from T98G and U87MG treated with both drug combinations (CXB+TMZ and NS398+TMZ) was observed, suggesting that COXIB counteracted the increase of EV-COX-2 content induced by TMZ (**Figure 4B**).

### Intracellular Uptake of EV-COX-2 Protein in Recipient Human Macrophage Cell Line

**Figure 5:**
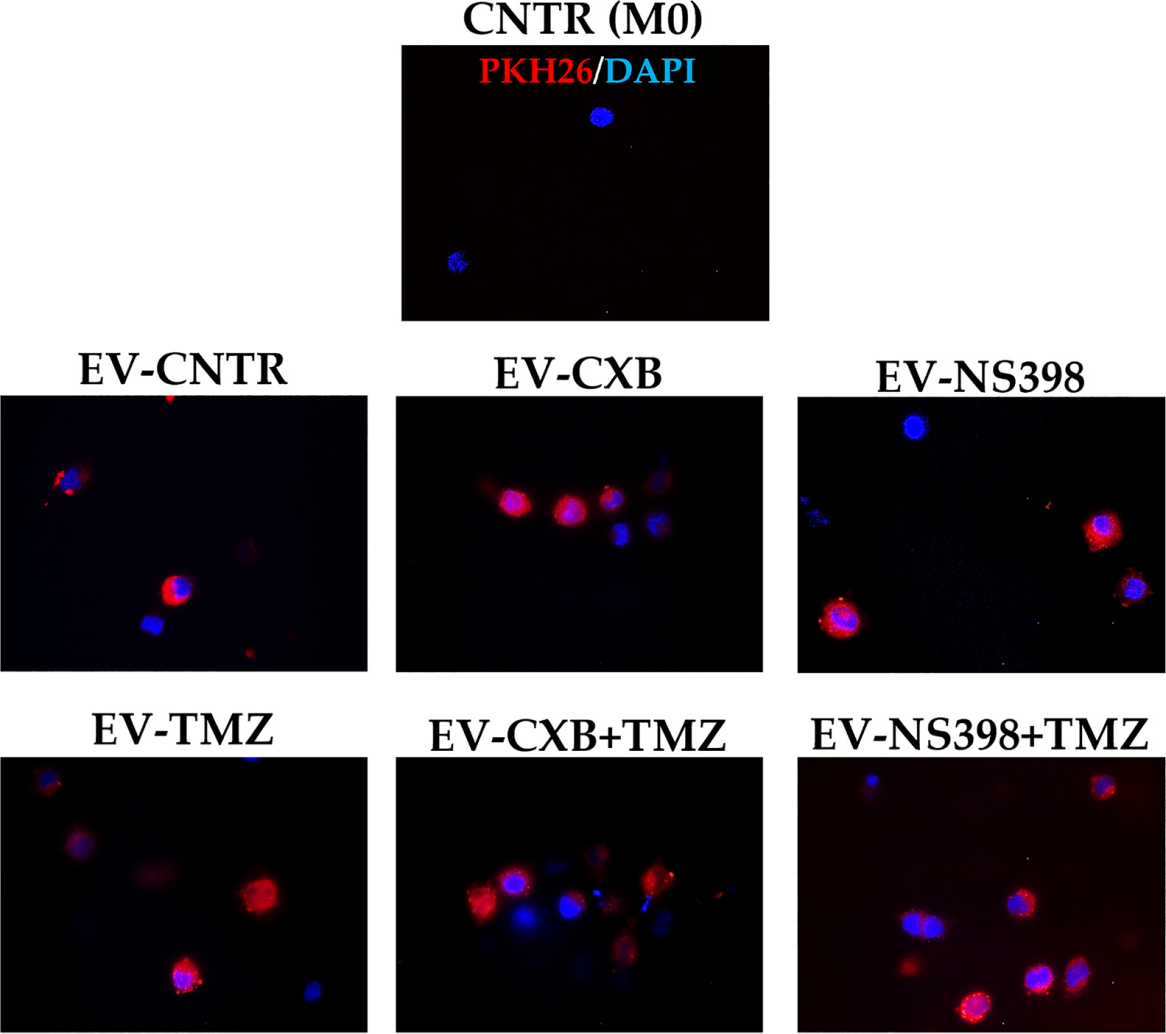
Macrophage U937 intracellular uptake of EV derived from control (CNTR) and CXB-, NS398-, TMZ-, drugs combination-treated T98G assessed by PKH26 staining. PKH26-labeled EV-COX-2 (red) were incubated at 30 μg/ml with U937 cells for 18 h. The nuclei of U937 were counterstained with DAPI (blue). CNTR (M0) = U937 cells not exposed to EV. Representative immunofluorescence images are from one out of three independent experiments. All images were acquired at 100× magnification.

It is also known that EV can mediate the communication in TME promoting tumor escape (34). Herein, we investigated the hypothesis that EV from TMZ-resistant T98G treated with COXIB- and TMZ, alone or in combination, could directly influence macrophage polarization. The internalization of T98G-EV by the U937 macrophage cell line was verified by PKH26 red fluorescent staining. Non-polarized U937 macrophages (M0) were incubated for 18 h with 30 μg/ml of PKH26-labeled EV derived from T98G exposed to COXIB, TMZ, and their mixture, as previously stated. U937 cells were permissive to PKH26-labeled EV entry. When PKH26-labeled EV were incubated with human U937 cells, we observed an effective uptake into the cytoplasm of all recipient cells, as indicated by red fluorescence (**Figure 5**).

**Figure 6:**
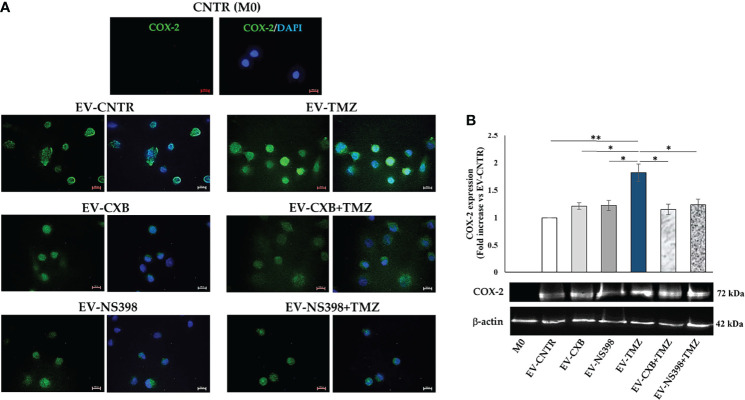
COX-2 levels in human macrophage cells after T98G-EV internalization. U937 macrophage cells (M0) were incubated with 30 μg/ml of EV from T98G daily treated in the absence (CNTR) or presence of COX-2 inhibitors CXB (8 μM), NS398 (20 μM), TMZ (10 μM) and their combinations for 5 days. **(A)** Representative immunofluorescence images from one out of two independent experiments showed the COX-2 (green) transferred by T98G-EV in U937 cells. Nuclei were counterstained with DAPI (blue). No green fluorescent signal was detected in the CNTR (M0) sample (U937 cells not exposed to EV). All images were acquired at 100× magnification (Scale bar = 10 µm). **(B)** COX-2 levels were analyzed by immunoblotting assay. Densitometric analysis was performed by normalizing vs. β-actin and expressed as fold increase vs. EV-CNTR. Data are from two independent experiments (mean ± SEM). For comparative analysis of data, a one-way ANOVA with *post hoc* Tukey test was used (*P<0.05, **P<0.01).

Therefore, COX-2 protein levels were evaluated in recipient U937 cells (M0) following exposure to EV derived from T98G treated as above described. Fluorescence images revealed the presence of COX-2 protein in macrophage (M0) appearing diffuse throughout the cytoplasm (green fluorescence). Macrophages M0 (COX-2 negative) not exposed to T98G-EV were used as a negative control. A significant COX-2 presence in U937 exposed to EV from TMZ-T98G cells was observed. Of interest, a lower COX-2 content was detected in U937 incubated with EV-CXB+TMZ and EV-NS398+TMZ compared to EV-TMZ (**Figure 6A**). The COX-2 quantification by Western blot analysis showed a significant increase in COX-2 level when U937 were exposed to EV-TMZ (**Figure 6B**). On the other hand, COX-2 levels in U937 exposed to EV-COXIB+TMZ were comparable to those of U937 exposed to EV-CNTR (**Figure 6B**).

### Effect of EV from T98G Treated With COXIB in Combination With TMZ on Macrophages

**Figure 7:**
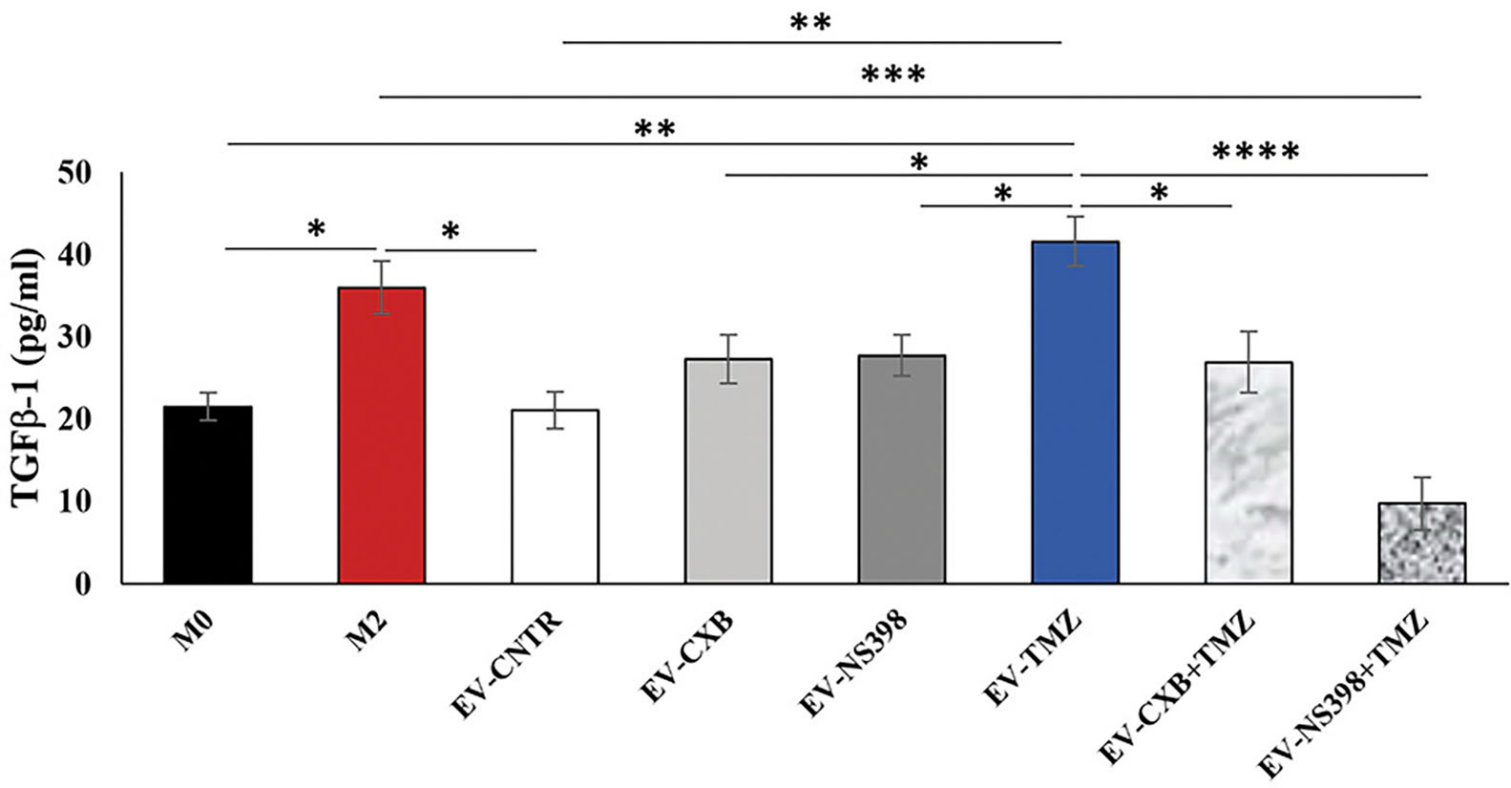
Effect of EV from treated-T98G on U937 macrophage cell line. The macrophage U937 cells (M0) were incubated with EV derived from T98G treated in the absence (CNTR) or presence of COXIB, CXB (8 μM), NS398 (20 μM), TMZ (10 μM) and their combinations for 5 days. Levels of TGF-β1, M2-related marker, were analyzed by ELISA kit in the cell supernatants of U937 exposed to EV from T98G. Macrophage M2 polarization was obtained by incubation with 20 ng/ml of interleukin 4 (IL-4) and 20 ng/ml of interleukin 13 (IL-13) for 72 h. The results relative to three experiments in duplicate are expressed as mean ± SEM. For comparative analysis of data, a one-way ANOVA with Tukey *post hoc* test was used (*P<0.05; **P<0.01; ***P<0.001).

GBM is sustained by a complex and highly immunosuppressive TME also responsible for therapy resistance. In this context, we finally investigated whether the EV secreted by T98G treated with COXIB, TMZ, and respective combinations, containing different levels of COX-2, were able to induce a phenotype modulation in the recipient U937 cells. To verify the macrophage M2 polarization after T98G-EV internalization, TGF-β1 released in the culture medium was assayed. M2 polarized macrophages, obtained as described in the materials and methods section, were used as the positive control. The extracellular levels of TGF-β1 released by U937 cells significantly increased after exposure to EV from TMZ-treated T98G (EV-TMZ) when compared to EV from CNTR (P<0.05) (**Figure 7**). No effect was observed when U937 cells were exposed to EV derived from T98G treated with single COXIB. In contrast, the TGF-β1 level was significantly lower in the supernatants of U937 treated with EV-COXIB+TMZ than EV-TMZ (**Figure 7**).

### Effect of EV Released from TMZ-Treated T98G Cells on U251MG

**Figure 8:**
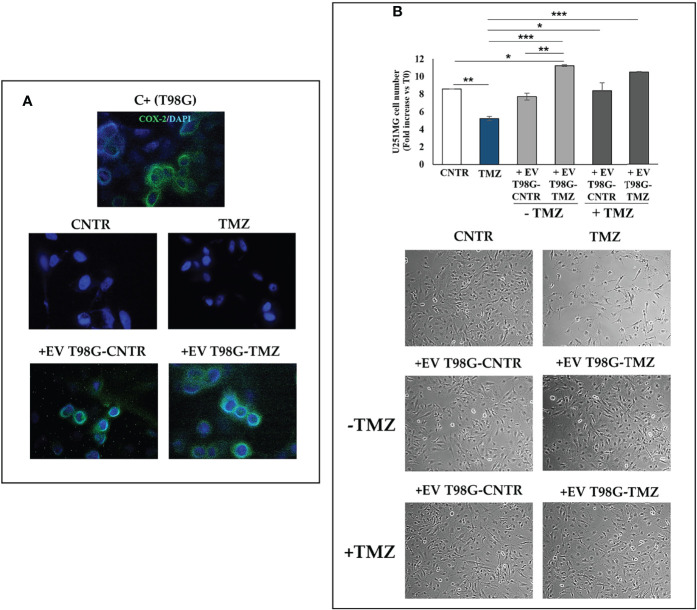
Effect of EV released from TMZ-treated T98G cells on U251MG TMZ-sensitive cells. The U251MG cells were incubated in presence or absence (CNTR) of TMZ (10 μM) or with 30 μg/ml EV derived from T98G treated with or without TMZ (10 μM) for 5 days**. (A)** Representative immunofluorescence images of U251MG cells treated or not as above described and stained with anti-COX-2 antibody (green) from one out of two independent experiments are shown. Nuclei were counterstained with DAPI (blue). T98G cells are used as positive control (C+). All images were acquired at 100× magnification. **(B)** Cell number was recorded by Trypan blue staining and representative images were taken at 10× magnification. All values are given as fold increase vs. initial time (T0). The results, derived from three experiments performed in duplicate, are expressed as mean ± SEM. For comparative analysis of data groups, a one-way ANOVA with Tukey *post hoc* test was used (*P<0.05; **P<0.01; ***P<0.001).

To further explore the role of EV-COX-2 in the TMZ resistance, we examined the cell growth of U251MG recipient cells, exposed to EV secreted by T98G treated or not with TMZ (10 µM). The EV internalization in U251MG target cells was allowed by incubation for 18 h. Representative images of immunofluorescence staining of U251MG cells treated with EV secreted from T98G cells showed the COX-2 delivery in the recipient cells, as detectable by green fluorescence (**Figure 8A**). Of note, in U251MG exposed to EV from TMZ-T98G, the fluorescence appeared more intense than relative CNTR according to TMZ’s ability to upregulate shuttled-COX-2 levels. No green fluorescence was detected in U251MG treated with or without TMZ.

As expected, TMZ treatment significantly reduced the U251MG cell growth versus CNTR (P<0.01) (**Figure 8B**). The uptake of EV from T98G-CNTR did not influence the cell number with respect to U251MG CNTR. Differently, the internalization of EV T98G-TMZ significantly increased the U251MG growth rate when compared to CNTR (P<0.05) and TMZ (P<0.001) (**Figure 8B**). Of note, when the U251MG were exposed to TMZ for an additional 72 h, the cell growth did not decrease in cells that have previously internalized the EV derived from CNTR and TMZ-treated T98G, showing a similar trend to those without TMZ treatment. Therefore, the EV derived T98G were able to strongly hinder the chemosensitivity. Representative images from microscopic observations confirmed these results (**Figure 8B**).

## Discussion

In this work, we show evidence that low and clinically relevant TMZ concentrations, able to upmodulate COX-2 in T98G and U87MG cells, led to a dose-dependent increase of the COX-2 levels in secreted EV. It is fair to point out that the results obtained with repeated exposure to TMZ for 5 consecutive days at clinically relevant concentrations, showing the TMZ-induced COX-2 increase as well as the increased sensitivity to TMZ in the presence of COXIB, have the same trend as those obtained in our previous work (8) where high doses of TMZ for 72 h were used on TMZ-resistant cells. Hence, the similarity between the effects of metronomic low dose application and those of single high dose protocol could slightly mitigate the criticalities raised toward *in vitro* studies in which the use of un-physiological high TMZ concentrations is questioned since it does not faithfully reproduce the clinical situation.

The ability of TMZ to induce an upregulation of COX-2 expression could be ascribed to the action exerted by the drug at the EGF/EGFR pair level leading to the NF-κB transcription factor activation able to upregulate the COX-2 expression due to the presence in the COX-2 promotor of the NF-κB response element (35). The NF-kB/COX-2 signaling is a crucial regulator of the malignant phenotype and chemoresistance in GBM (36). NF-κB signaling pathway has also been reported to influence radiotherapy tolerance of glioma cells through regulating COX-2 expression, with potential therapeutic approaches for the treatment of glioma (37).

The TMZ-induced increase of COX-2 in EV was significantly counteracted by the treatment of the cells with COXIB in combination with TMZ. The EV derived from T98G exposed to drug combination treatment were able to prevent the TGF-β1 release by recipient macrophage cells induced by EV secreted by TMZ-treated T98G, thus neutralizing the M2-polarization.

Experimental studies carried out in recent years on the EV derived from GBM have revealed numerous molecules in their cargo (38); however, to the best of our knowledge, the presence of COX-2 protein in EV derived from GBM cells has never been verified. In 2017, Kim and colleagues (39) reported the transfer of the COX-2 protein by exosomes from COX-2-positive lung cancer cell lines affecting the phenotype of monocytes THP-1, used as recipient cells. The COX-2 uptake by THP-1 determined an increased production of PGE2 and VEGF sustaining tumor growth. In our previous study, the addition of NS398 caused a functional change of EV released by GBM stem cells which, in turn, provoked a decrease in cell migration and autophagy induction in adherent U87MG and T98G, used as recipient cells (40).

The neuroinflammation and the role of inflammatory mediators, such as COX-2, are critical components in establishing an immunosuppressed microenvironment, thus fueling GBM proliferation, invasion, and maintenance of stemness features (41). COXIB enhanced the tumor-associated-macrophage-mediated anti-tumor immune responses by increasing monocyte cytokine production (42). The COX-2 role in macrophage polarization was also analyzed after NS398 treatment of bone marrow-derived macrophages that increased the secreted levels of TNFα and reduced the IL-10 secretion (42). Our present findings show that EV released by TMZ-treated T98G shuttled COX-2 and, after effective internalization by U937 macrophage cells, induced a higher level of TGF-β1, a hallmark of the transition in M2 macrophage state (43). Of interest, the EV-COX-2 made the recipient U251MG, TMZ-sensitive cells, less responsive to TMZ action, suggesting a possible role of EV derived from TMZ-resistant cells in the transfer of chemoresistance through the COX-2 delivering.

**Figure 9:**
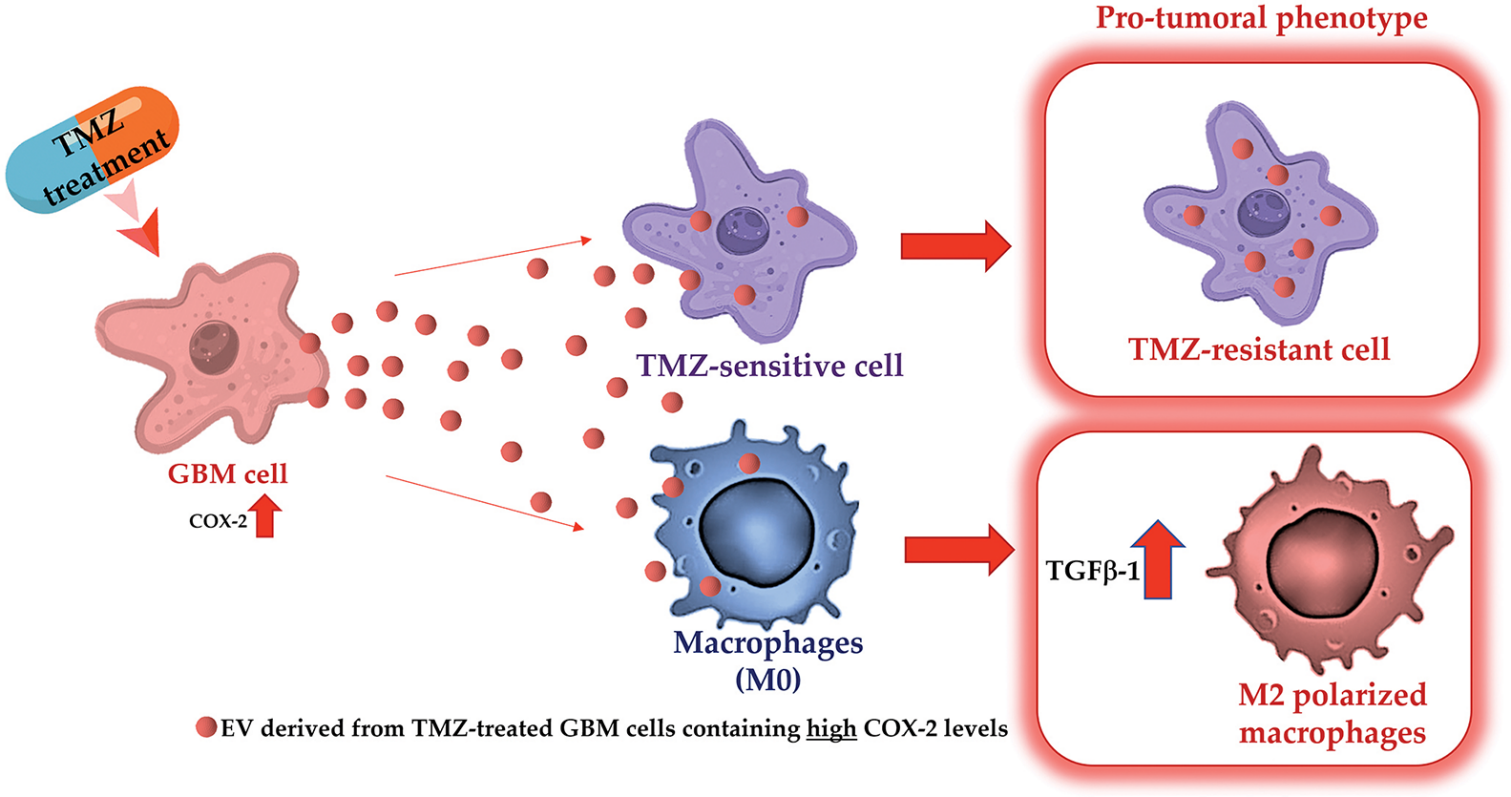
Graphical representation of experimental design and main obtained results.

Overall, the results suggest that COX-2 shuttled by EV can modulate the fate of cells in the GBM microenvironment making them less sensitive to the alkylating drug action. **Figure 9** shows a graphical representation of the main results.

Although our data represent the first evidence of the presence and effective functional transfer of COX-2 through EV derived from GBM cells, further and more comprehensive studies are needed on the multiple consequences at the level of TME of the presence of COX-2 in EV. Likewise, it will be essential to deepen COXIB’s effects on tumor cells and the TME. Although many questions remain unanswered regarding the precise molecular and cellular mechanisms involved in GBM resistance, strategies to modulate the TME can offer a new perspective on the clinical approach.

We highlighted a possible scenario to counteract the TMZ-resistance using COXIB to modulate the GBM neuroinflammation and immunosuppression in the TME through EV and enhance the TMZ therapeutic effectiveness. Our data support a combined therapeutic strategy of administering COXIB and TMZ to modulate the content of local EV with the aim of improving the response to chemotherapy in GBM patients.

## Data Availability Statement

The raw data supporting the conclusions of this article will be made available by the authors, without undue reservation.

## Author Contributions

MGC, BC, and PP contributed to conception of the study; FL, FRA, SA, and PP performed the experimental work. IG and VD performed the EV isolation and characterization. FL, FRA, IG, PP performed data analyses. PP and FL performed the statistical analysis of data, wrote the original manuscript, and produced all figures. MGC, EA, VD, BC, and PP performed the revision and editing of the manuscript and provided overall guidance for the experiments. FL, MGC, BC, and PP provided for the founding acquisition. All authors listed contributed to final manuscript revision and agreed to the published version of the manuscript.

## Funding

This research was funded by Department of Life, Health & Environmental Sciences, University of L’Aquila, grant number 822/2021 “Fondi Bando Ricerca FFO 2021” and grant number 786/2021 “Progetti di Ateneo per la ricerca di base e avvio alla ricerca 2021–2022” and grant number “Bando PSD-MESVA 2022”.

## Conflict of Interest

The authors declare that the research was conducted in the absence of any commercial or financial relationships that could be construed as a potential conflict of interest.

## Publisher’s Note

All claims expressed in this article are solely those of the authors and do not necessarily represent those of their affiliated organizations, or those of the publisher, the editors and the reviewers. Any product that may be evaluated in this article, or claim that may be made by its manufacturer, is not guaranteed or endorsed by the publisher.

## Supplementary Material

Supplementary Figure 1 Effect of TMZ on GBM cell viability. Representative phase-contrast images (10× magnification) of **(A)** T98G and **(B)** U87MG in the absence (CNTR) or presence of TMZ at several concentrations for 5 days are shown.

Supplementary Figure 2 Influence of TMZ on **(A)** COX-2, **(B)** β-catenin and **(C)** MGMT expression in U251MG TMZ-sensitive cell line. Immunoblotting assays were performed on cells incubated for 5 days in the presence or absence (CNTR) of TMZ (5-20 µM). β-actin serves as internal control. The images are representative of three independent experiments. **(D)** Influence of the COXIB combined with TMZ on COX-2 levels was verified by Western blotting assay in U251MG cells daily incubated or not (CNTR) with Celecoxib (CXB) (8 μM), NS398 (20 μM), TMZ (10 μM) or with the co-treatments (CXB+TMZ and NS398+TMZ) for 5 days. Representative images of each immunoblotting are shown. C+ = positive control (not treated T98G).
