# Supplementary material for: Cyclooxygenase-2 Upregulated by Temozolomide in Glioblastoma Cells Is Shuttled In Extracellular Vesicles Modifying Recipient Cell Phenotype
Source: Front Oncol. 2022 Jul 22;12:933746. doi: 10.3389/fonc.2022.933746 (PMC9355724; doi:10.3389/fonc.2022.933746)

Cyclooxygenase-2 upregulated by temozolomide in glioblastoma cells is shuttled in extracellular vesicles modifying recipient cell phenotype

Francesca Lombardi, Francesca Rosaria Augello, Serena Artone, Emira Ayroldi, Ilaria Giusti, Vincenza Dolo, Maria Grazia Cifone, Benedetta Cinque and Paola Palumbo

Supplementary Figure 1: Effect of TMZ on GBM cell viability. Representative phase-contrast images (10× magnification) of **(A)** T98G and **(B)** U87MG in the absence (CNTR) or presence of TMZ at several concentrations for 5 days are shown.

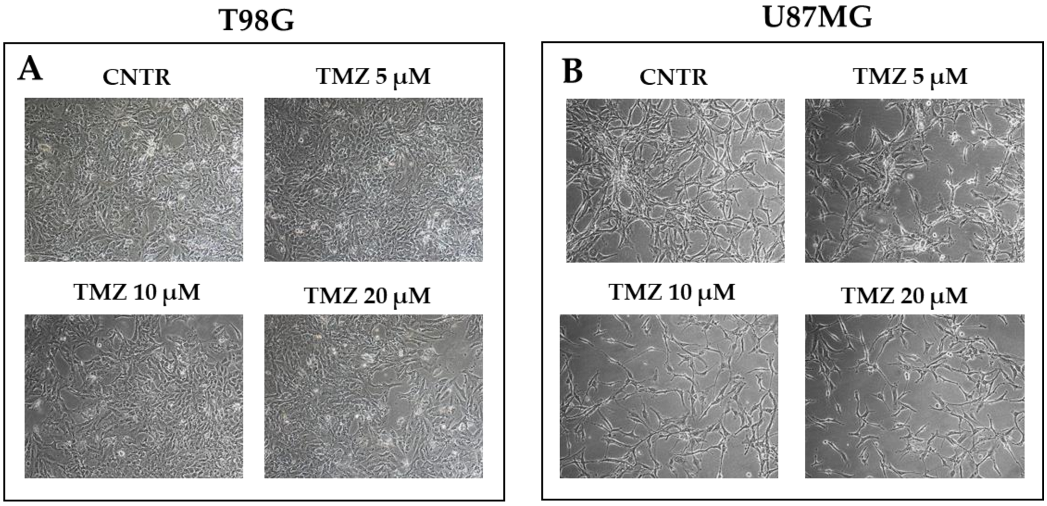

Supplementary Figure 2: Influence of TMZ on **(A)** COX-2, **(B)** β-catenin and **(C)** MGMT expression in U251MG TMZ-sensitive cell line. Immunoblotting assays were performed on cells incubated for 5 days in the presence or absence (CNTR) of TMZ (5-20 μM). β-actin serves as internal control. The images are representative of three independent experiments. **(D)** Influence of the COXIB combined with TMZ on COX-2 levels was verified by western blotting assay in U251MG cells daily incubated or not (CNTR) with Celecoxib (CXB) (8 μM), NS398 (20 μM), TMZ (10 μM) or with the co-treatments (CXB+TMZ and NS398+TMZ) for 5 days. Representative images of each immunoblotting are shown. C+ = positive control (not treated T98G).

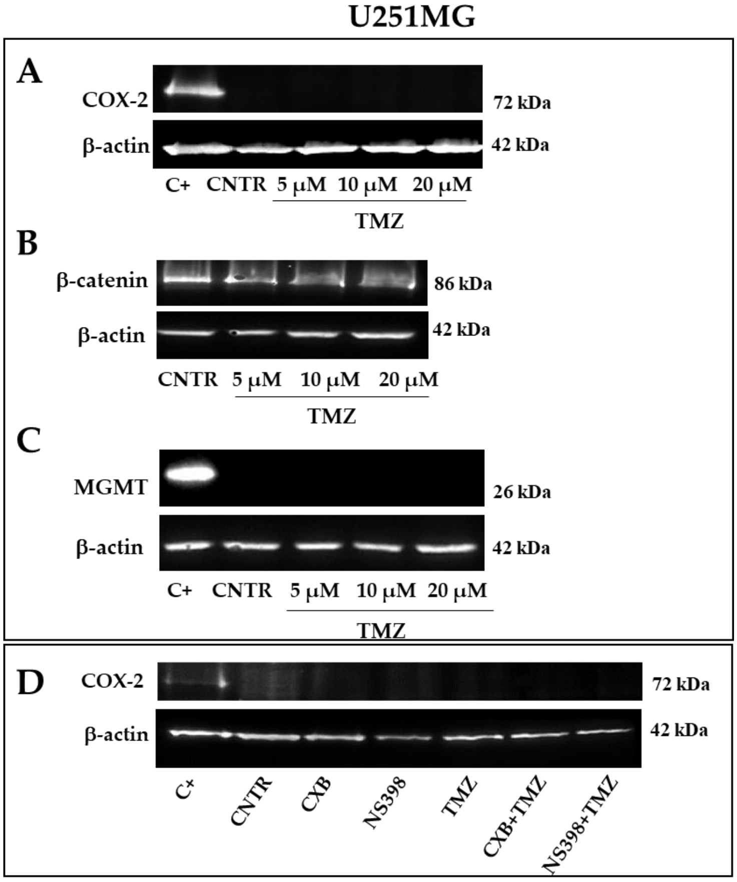

Supplement: Supplementary Figure 1 — Effect of TMZ on GBM cell viability. Representative phase-contrast images (10× magnification) of (A) T98G and (B) U87MG in the absence (CNTR) or presence of TMZ at several concentrations for 5 days are shown. [file DataSheet_1.pdf]
